# Rapid loss of a green fluorescent plasmid in *Escherichia coli* O157:H7

**DOI:** 10.3934/microbiol.2017.4.872

**Published:** 2017-10-25

**Authors:** Anil K. Persad, Michele L. Williams, Jeffrey T. LeJeune

**Affiliations:** 1 Food Animal Health Research Program, Ohio Agricultural Research and Development Center, The Ohio State University, Wooster, Ohio, USA

**Keywords:** GFP plasmid, rifampicin, survival studies, marker stability, *Escherichia coli* O157:H7

## Abstract

Plasmids encoding green fluorescent protein (GFP) are frequently used to label bacteria, allowing the identification and differentiation from background flora during experimental studies. Because of its common use in survival studies of the foodborne pathogen *Escherichia coli* O157:H7, it is important to know the extent to which the plasmid is retained in this host system. Herein, the stability of a pGFPuv (Clontech Laboratories Inc) plasmid in six *Escherichia coli* O157:H7 isolates was assessed in an oligotrophic environment (phosphate buffered saline, PBS) without antibiotic selective pressure. The six test isolates were recovered from a variety of animal and human sources (cattle, sheep, starlings, water buffalo, and human feces). GFP labeling of the bacteria was accomplished via transfer electroporation. The stability of the GFP plasmid in the different *E. coli* O157:H7 isolates was variable: in one strain, GFP plasmid loss was rapid, as early as one day and complete plasmid loss was exhibited by four of the six strains within 19 days. In one of the two isolates retaining the GFP plasmid beyond 19 days, counts of GFP-labeled *E. coli* O157:H7 were significantly lower than the total cell population (*P* < 0.001). In contrast, in the other isolate after 19 days, total *E. coli* O157:H7 counts and GFP-labeled *E. coli* counts were equivalent. These results demonstrate strain-to-strain variability in plasmid stability. Consequently the use of GFP-labeled *E.coli* O157:H7 in prolonged survival studies may result in the underestimation of survival time due to plasmid loss.

## 1. Introduction

Shiga toxin-producing *Escherichia coli* (STEC), a diverse group of bacteria with over 400 serogroups, is estimated to cause over two million cases of human disease globally each year. Historically, STEC O157:H7 has been the pathotype most associated with human disease, with clinical signs ranging from mild to bloody diarrhea, with approximately 4% of adult cases and 15% of children [1] progressing to more severe clinical syndromes such as hemolytic uremic syndrome (HUS). Mortality rate can be up to 3%, and 70% of cases recover fully within 5 years however, but the remainder can suffer lifelong neurologic and kidney function deficits [2]. Understanding the survival of *E. coli* O157:H7 in the environment is useful to assess health risks associated with environmental contamination. To determine the survival of an organism in the environment, one must be able to identify the bacteria of interest and accurately estimate its population size, often in the presence of background microbial flora. Unique genotypic and/or phenotypic traits can be exploited to facilitate the enumeration of target organisms admixed with background organisms. In nature however, unique markers are not always naturally occurring. Genetic manipulations of organisms of interest, either via transformation or chromosomal insertions, deletions or mutations, are useful laboratory methods to label bacteria. Two popular phenotypic characteristics that allow easy identification and differentiation include the use of antibiotic resistance and/or fluorescence.

The green fluorescent protein (GFP) genes which encode for the fluorescing protein were originally isolated from the jellyfish *Aequorea victoria* [3]. These proteins fluoresce when exposed to UV light with maxima excitation and emission occurring at wavelengths of 395 nm and 509 nm respectively. Exposure to UV light within this spectrum can thus be used to identify bacteria expressing GFP. Many of the plasmids encoding this protein have been engineered to also contain genes which encode for antibiotic resistance [4]. The presence of these plasmids has been reported to have no effect on the biochemical, morphological, or survival characteristics of the bacterial cells labeled with them [5]. The use of plasmids encoding GFP provide the advantage of allowing ease of detection with minimal additional processing and without the need for agar supplementation for metabolic selection [6], which can be detrimental to the recovery of sublethally injured organisms [7]. However, since plasmids are extra-chromosomal DNA molecules, they can be lost from the cell via spontaneous segregation and deletion during replication, exposure to high temperatures, nutrient deprivation, or the use of curing agents [8]. In long term survival experiments, it is essential that this marking mechanism be stable for the duration of an experiment and not be lost. Failure of the *E. coli* O157:H7 to retain the plasmid will result in loss of the marking mechanism, and consequently under-estimation of colony counts and bacterial survival and herein lies the importance of our study.

The purpose of this study was to evaluate the stability of a GFP plasmid in *E. coli* O157:H7 when removed from an antibiotic selective environment and placed in an oligotrophic environment similar to that which would be encountered during a prolonged environmental survival study. In addition, we compared the recovery of these GFP-labeled *E. coli* O157:H7 strains with the stability of a chromosomally marked *E. coli* O157:H7 in a similar environment.

## 2. Materials and Method

### 2.1. Bacterial strains and labeling of *E. coli* O157:H7

The stability of the pGFPuv plasmid (Clontech Laboratories Inc., Mountain View, CA) in six epidemiologically unrelated *E. coli* O157:H7 isolates recovered from the feces of five different animal species was studied. For comparison, we also compared the stability of one chromosomally labelled *E. coli* O157:H7 isolate. The test isolates were recovered from the feces of cattle, water buffalo, sheep, starlings, and humans (Table 1). Transformation was attained via electroporation using the Gene Pulser 2 (BioRad, CA) in a traditional fashion as described by Sambrook et al. [9].

**Table 1.** Identification and origin of *E.coli* O157:H7 strains used in this study.

Strain Number	Original Name	Species of Origin	Reference
EC 22	c363-83	Water buffalo (*Bubalus bubalis*)	[10]
EC 811	EC 811	Beef cattle (*Bos taurus*)	[11]
EC 1259	EC 1259	Human (*Homo sapiens*)	[12]
EC 1513	EC 1513	Sheep (*Ovis aries*)	[13]
EC 1628	EC 1628	Starling (*Sturnus vulgaris*)	[14]
EC 1767	EC 4042	Human (*Homo sapiens*)	[15]

Test strains were recovered from frozen stock and transformed with a GFP-encoding plasmid. Briefly, to obtain clonal populations of bacteria, stock cultures frozen at −80 °C in 30% buffered glycerol solution were allowed to thaw and one loopful of stock was streaked onto a sorbitol MacConkey agar (SMACct) plate containing cefixime (50 ng/mL; Invitrogen Dynal A.S., Oslo, Norway) and potassium tellurite (2.5 µg/mL; Invitrogen Dynal A.S.). Plates were incubated overnight at 37 °C. The next day, one colony was selected and used to inoculate a 50-ml centrifuge tube containing 10 ml of LB-Lennox broth (Acumedia, MI). This was then incubated at 37 °C for 24 hours while being shaken at 110 rpm. After incubation, 1 ml of this broth was used to inoculate a flask containing 30 ml LB broth. This flask was then placed on a shaker and incubated at 35 °C until an OD_600_ of 0.6 was attained. The contents of the flask were then transferred to a 50-ml centrifuge tube and contents centrifuged at 4600 × g for 20 minutes at 4 °C. The supernatant was then decanted and the pellet resuspended with 25 ml of sterile water. This wash procedure was repeated two more times. After the third washing, the pellet was resuspended in 10 ml of sterile water. Forty microliters of this washed cell culture was mixed with 1 µl of plasmid DNA (59.9 ng/µl) and incubated on ice for 1 min. The sample was placed in a 0.1 cm electroporation cuvette and subjected to an electric pulse (1.8 kV, 200 Ω, 25 µF). The cells were then recovered by incubation in non-supplemented LB-Lennox broth at 37 °C for 2 hours with shaking on an orbital shaker. After incubation, the sample was plated on LB-Lennox agar (Acumedia, MI) supplemented with 100 µg/ml ampicillin (Fisher Scientific, NJ) and incubated at 37 °C overnight. Three fluorescing transformants were then selected visually based on their fluorescence under UV light, cultured in brain heart infusion (BHI) broth (Acumedia, MI), and stored at −80 °C with 30% buffered glycerol (VWR International, OH).

Chromosomal marking of *E.coli* O157:H7 (EC 1767) was achieved by selecting for naturally occurring resistance to rifampicin resistance. Briefly, the parent *E. coli* O157:H7 isolate was propagated overnight at 37 °C in tryptic soy broth (Acumedia, MI) and 100 µl of this broth was transferred daily to fresh media with increasing concentrations (10, 20, 30, 40, 50, 60, 70, 80, 90, 100 µg/ml) of rifampicin (Fisher Scientific). The final broth culture was plated on LB agar with rifampicin (80 µg/ml) and incubated overnight at 37 °C. Three isolates recovered from the LB plates were grown overnight in BHI broth and stored at −80 °C with 30% buffered glycerol for use at a later time.

### 2.2. Short-term growth kinetics of labeled bacteria and wild-type *E.coli* O157:H7 parent

The short-term growth kinetics of the labeled strains and the parent strains were compared over a 10-hour period. Three transformed GFP-labeled isolates from each of the five strains were recovered from frozen stock and streaked for isolation on LB-Miller agar (Acumedia, MI) supplemented with 100 µg/ml ampicillin (LBAP100). One colony from each culture was then used to inoculate separate 15-ml centrifuge tubes containing 5 ml of LB-Lennox broth (Acumedia, MI) supplemented with 100 µg/ml ampicillin. These inoculated tubes were then incubated at 37 °C for 24 hours while being shaken at 110 rpm. The next day, 1 ml of each broth was used to inoculate separate flasks containing 500 ml of LB-Lennox broth. The flasks were then placed on a shaker (110 rpm) and incubated at 37 °C until time of sampling. Every hour, 1ml from each the three broth cultures was diluted 1-fold in phosphate buffered saline (Amresco, OH) and spread plated using sterile glass beads on LB agar. Plates were incubated for 24 hours at 37 °C and the concentration of bacteria in the broth determined for each sample period.

### 2.3. Validation of labeled strains in long-term survival study

The GFP labeled isolates were streaked from frozen stock for isolation on LBAP100. One colony from each isolate was then used to inoculate separate 15 ml centrifuge tubes containing 5 ml of LB broth supplemented with 100 µg/ml ampicillin. These inoculated tubes were then incubated at 37 °C for 24 hours while being shaken at 110 rpm. The next day, 1 ml of each broth was used to inoculate separate flasks containing 250 ml of LB-Lennox broth supplemented with 100 µg/ml ampicillin and incubated for 16 hours at 37 °C on an orbital shaker (110 rpm). Forty-five ml samples of each inoculum were then placed in 50-ml centrifuge tubes and centrifuged at 4600 × g for 20 minutes. The supernatant was decanted and the bacterial pellet re-suspended in 45 ml of 1× PBS. This wash procedure was repeated twice to remove antibiotic and nutrient content from the inocula. After the third washing and re-suspension of the bacterial pellet in PBS, the washed inocula were aliquoted into 1 ml volumes in 2-ml micro centrifuge tubes and stored at 37 °C until time of sampling. This was done in triplicate.

A similar method to that described above was used to obtain a rifampicin-resistant *E. coli* O157:H7 inoculum devoid of nutrient and antibiotic selection pressure. The washed inocula was then also separated into 1 ml aliquots and stored at 37 °C until time of sampling.

Plasmid stability was assessed daily. Each of the three replicate PBS 1-ml survival microcosms was serially diluted with PBS up to a dilution of 10^−6^. One hundred µl samples of each dilution were spread plated on LB-Miller agar using sterile glass beads. These plates were incubated at 37 °C for 24 hours. The number of total colonies on a plate was counted and aided by UV transillumination, the number of fluorescent colonies present was enumerated. Using plates yielding between 20 and 300 colonies, the fraction of fluorescing and non-fluorescing colonies present on each day was calculated. Each day, up to ten (if present) non-fluorescent colonies were tested to confirm the presence of the O157 antigen using a latex agglutination test (Oxoid, Kent, UK) and cultured on LBAP100 to confirm their loss of resistance to ampicillin.

Six non-fluorescent colonies were selected daily throughout the study to be tested to confirm the loss of the pGFPuv plasmid. Briefly, one non-fluorescing colony was used to inoculate 45 ml LB broth. This starter culture was incubated on an orbital shaker at 110 rpm for 24 hours at 37 °C. After incubation, 600 µl of the culture was transferred to a 1.5-ml microcentrifuge tube. The tube was then centrifuged (Microfuge 2, Beckman Coulter, CA) at 18,000 × g for two minutes at 4 °C. Thereafter, 550 µl of the supernatant removed ensuring the bacterial pellet was not disturbed. The bacterial pellet was then resuspended and 0.5 µl of RNaseA (20 mg/ml; Novagen, MA) and 40 µl of phenol/chloroform/isoamyl alcohol (25:24:1; Sigma, MO) were added to the tube and the contents thoroughly mixed. The contents were then centrifuged as described above and the supernatant transferred to a sterile 2-ml microcentrifuge tube. The supernatant was subjected to gel electrophoresis on a 0.7% agarose gel (AMRESCO, OH). The same procedure was repeated as outlined above for the transformed fluorescing *E. coli* O157:H7.

For comparative purposes, an evaluation of the stability of a chromosomally marked *E. coli* O157:H7 was assessed weekly. Ten-fold serial dilutions from each of the three replicate PBS 1-ml survival microcosms were made in PBS up to a dilution of 10^−6^. One hundred µl samples of each dilution was spread plated using sterile glass beads on LB-Miller agar and also on LB-Miller agar supplemented with 80 µg/ml rifampicin. The number of colonies present on each plate was counted after 24 h incubation at 37 °C. Ten colonies from the LB-Miller agar plate were selected weekly and subjected to an *E. coli* O157 latex agglutination test to confirm the presence of the O157 antigen.

### 2.4. Statistical analysis

Plasmid burden was evaluated by fitting growth data to the Gompertz model [16] and model coefficients for the lag time, the maximum growth rate, and the maximum asymptote for the GFP-labeled cells and their respective parent were generated. A t-test was used to evaluate any statistical differences between the lag time for the parent and the labelled isolates. The maximum growth rate and the asymptote of the parent and the labelled isolates were similarly evaluated for any statistically significant difference. To evaluate the stability of the labelling mechanism, a repeated measures ANOVA test (Minitab 16.0) was used to examine the differences between the total cell count and the number of fluorescing cells. Likewise, a repeated measures ANOVA was also used to evaluate the stability of the chromosomally labeled *E. coli* mutants. Differences were considered to be statistically significant at *P* < 0.05.

## 3. Results

### 3.1. Short-term growth kinetic evaluation

**Figure 1.**
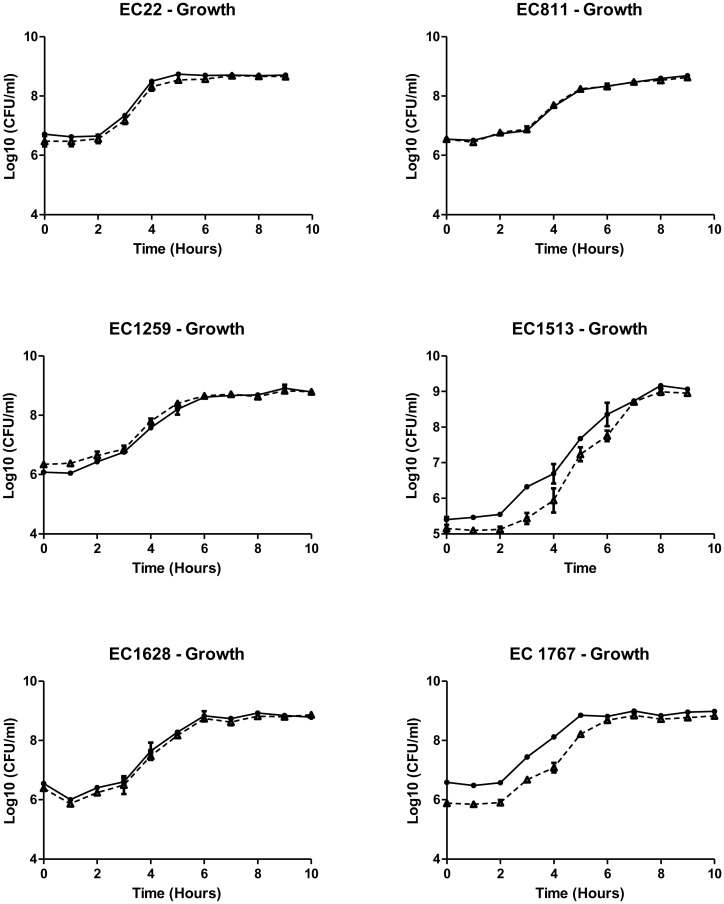
Comparison of growth rates of the parental *Escherichia coli* O157:H7 strains (•) and their respective of the GFP-labeled strains (▴). For all *E. coli* O157:H7 strains evaluated the growth of the GFP-labeled strains accurately reflected that of the parent strain (Table 2). Values are expressed as the mean Log10 CFU/ml of a minimum of two isolates ± standard error.

The evaluation of the short-term growth kinetics of the GFP plasmid labeled strains and Chromosomal strains and their respective *E. coli* O157:H7 parent strains revealed no significant differences. Lag periods and maximum growth rates and asymptote values for both the GFP plasmid labeled *E. coli* O157:H7, and the chromosomally marked rifampicin resistant *E. coli* O157:H7 were similar to their respective parents (Table 2, Figure 1).

**Table 2.** Comparison of growth coefficients for parent and labeled isolates determined using Gompertz model.

Isolate Number	Isolate	Lag Time (hours)	*P* Value	Maximum Growth Rate	*P* Value	Maximum Asymptote (Log10 CFU/ml)	*P* Value
EC 22	Parent	3.05 ± 0.50	0.31	3.53 ± 0.23	0.98	8.70 ± 0.02	0.35
	GFP labeled	2.46 ± 0.11		3.51 ± 0.63		8.65 ± 0.04	

EC 811	Parent	2.53 ± 0.08	0.39	1.67 ± 0.01	0.38	8.68 ± 0.06	0.51
	GFP labeled	1.89 ± 0.57		1.40 ± 0.24		8.63 ± 0.02	
EC 1259	Parent	1.96 ± 0.17	0.31	1.76 ± 0.06	0.34	8.78 ± 0.07	0.95
	GFP labeled	2.37 ± 0.30		1.91 ± 0.13		8.79 ± 0.07	
EC 1513	Parent	2.68 ± 0.34	0.28	2.4 ± 0.25	0.25	9.06 ± 0.03	0.19
	GFP labeled	3.30 ± 0.35		3.26 ± 0.61		8.98 ± 0.04	
EC 1628	Parent	3.19 ± 0.25	0.14	2.96 ± 0.19	0.15	8.78 ± 0.04	0.16
	GFP labeled	2.85 ± 0.33		2.31 ± 0.31		8.86 ± 0.02	
EC 1767	Parent	2.22 ± 0.01	0.08	2.30 ± 0.01	0.34	8.98 ± 0.05	0.06
	Gfp labeled	2.28 ± 0.03		2.33 ± 0.06		8.82 ± 0.02	
EC 1767	Parent	3.25 ± 0.24	0.34	1.44 ± 0.08	0.19	8.49 ± 0.03	0.37
	Rif. labeled	3.12 ± 0.15		1.60 ± 0.11		8.45 ± 0.02	

Values listed are the Mean ± SE. There was no statistical difference in the growth kinetics parameters between the labeled *E. coli* O157:H7 strains and their respective parent strains.

### 3.2. Validation of labeled strains in a long-term survival study

**Figure 2.**
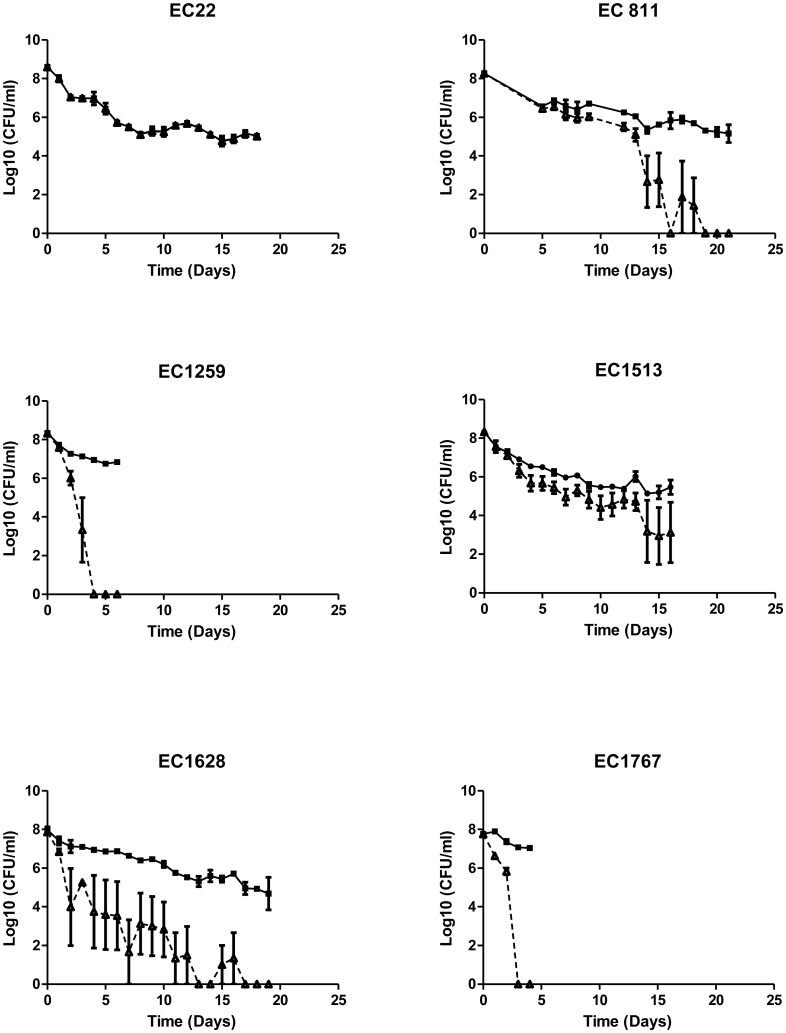
Stability of GFP-labeled *Escherichia coli* O157:H7 parent (▪) and respective GFP labelled isolates (▴). Only EC 22 and EC 1513 maintained the GFP plasmid for the duration of the experiments; for EC 1513 only, the GFP cell counts were statistically different from total cell count (*P* < 0.001). Values are expressed as the mean Log CFU/ml of minimum of two isolates ± standard error.

There was marked variability in the stability of the GFP plasmid-labeled *E.coli* O157:H7 isolates. The isolate recovered from the water buffalo (EC 22) maintained the GFP plasmid the longest (Figure 2) with the GFP-labeled isolate accurately reflecting the total cell count (number of fluorescing cells + non fluorescing cells) for the duration of the experiment (*P* = 0.818). The GFP plasmid was maintained for the shortest time in the two isolates recovered from human feces. For EC 1767, plasmid loss was almost 95% after one day and by the third day no fluorescing cells were recovered. The other isolate recovered from human feces, EC 1259, exhibited almost 95% plasmid loss by the third day and by day four no fluorescing cells were recovered. With the exception of EC 22, all of the other isolates tested began to exhibit plasmid loss at different time points during the experiment and using a repeated measures ANOVA, there was a statistically significant difference between the total cell counts and number of fluorescing cells (*P* < 0.001).

**Figure 3.**
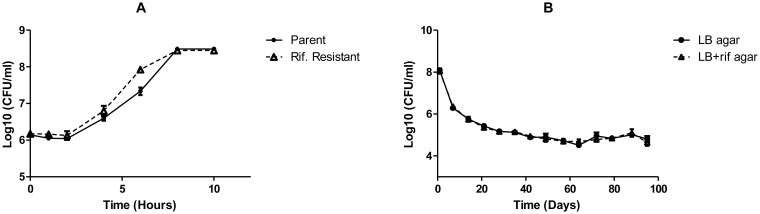
(A) Growth rate of parent and chromosomally labeled *E. coli* O157:H7 and (B) Stability of chromosomally marked rifampicin-resistant *E. coli* O157 in an oligotrophic, non-selective media. Rifampicin-labeled cell counts accurately reflected the total cell population for the duration of the experiment (*P* = 0.67). Values are expressed as the mean Log CFU/ml of minimum of two isolates ± standard error.

The chromosomally labeled *E. coli* O157:H7 exhibited a statistically similar lag time, maximum growth rate, and maximum asymptote to the parent isolate. For the duration of the experiment (Figure 3), there was no difference between the total cell count on the LB agar and the total cell count on the LB agar supplemented with rifampicin (80 µg/ml) (*P* = 0.67).

**Figure 4.**
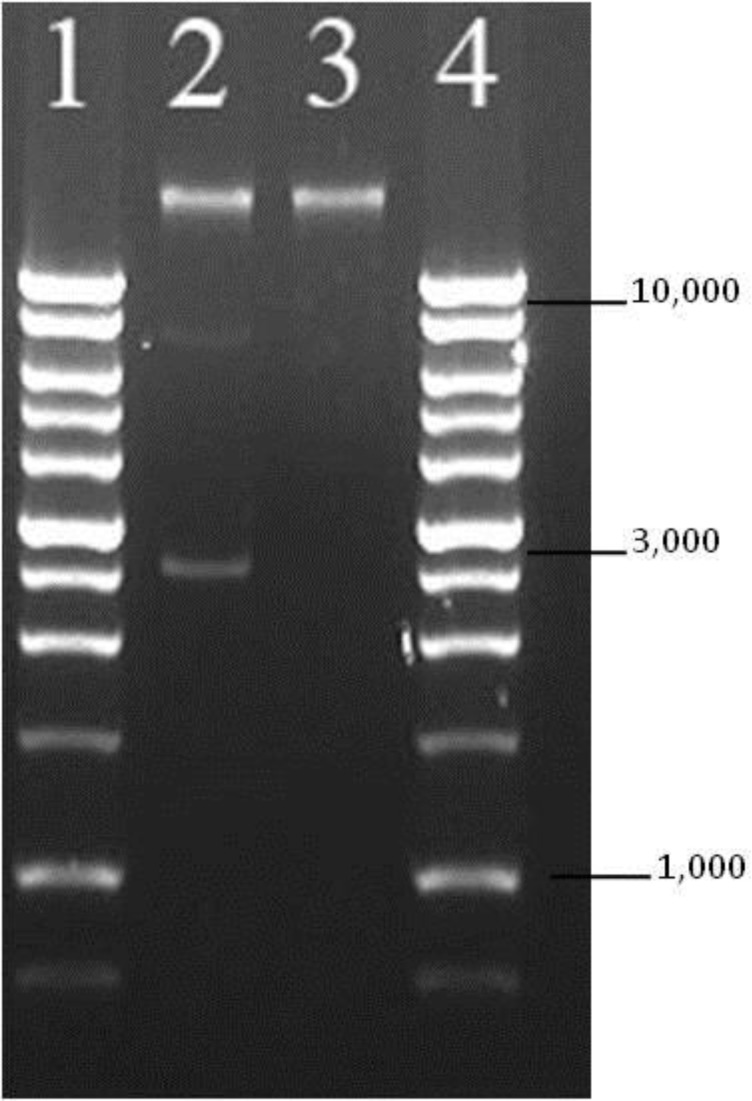
Electrophoretic gel demonstrating the location of the pGFPuv plasmid at the 3 kb region obtained from the GFP-labeled isolate (lane 2). In lane 3, a non-fluorescing O157:H7 isolate recovered during the study. There is no DNA band in this region indicating loss of pGFPuv plasmid. Lanes 1 and 4 contain the 1 kb DNA ladder (Promega, WI).

### 3.3. Plasmid screening and confirmation of plasmid loss

All of the randomly selected, non-fluorescent cells agglutinated with the anti-O157 latex agglutination test. Non-fluorescent colonies did not grow on media supplemented with ampicillin. The plasmid profiles of the representative fluorescent and non-fluorescent colonies were distinguishable; notably, the predicted 3 kb band in the fluorescent colony was absent in the ampicillin-susceptible, non-fluorescent colonies (Figure 4).

## 4. Conclusion

These experiments demonstrate that use of the fluorescent phenotype in GFP-labeled *E. coli* O157:H7 may not always accurately reflect the total culturable *E. coli* O157:H7 cells under low nutrient conditions in the laboratory. Of particular interest is that with some *E. coli* O157:H7 strains, despite the plasmid being maintained for short duration experiments, the plasmid and consequently the mechanism of bacterial differentiation were lost during long term experiments. Notably, the length of time the plasmid was retained seemed to be affected by the source of isolate and presumably the genetic background of the strain. The extent to which similar disparities in plasmid retention occur in other matrices and with other *E. coli* O157:H7 strains could have important implications on the interpretation of experimental survival studies that have used this method of marking. In contrast, the single chromosomally labeled *E. coli* O157:H7 which did not differ from its marked parent may provide a more suitable method of labeling *E. coli* O157:H7 for long-term survival studies since it maintained its phenotypic marker for the duration of the experiment. Additional survival studies of chromosomally marked *E. coli* O157:H7 strains are warranted.

The short-term stability of this plasmid in the *E. coli* O157:H7 population is consistent with previously published data [5],[17],[18],[19] and highlights the fact that it may be suitable for use in very short-term survival studies. The long term stability of the plasmid however has not been reported until now. This study demonstrates that although the plasmid may be maintained for short periods, for longer duration studies, the plasmid is lost. This loss of plasmid and consequently the visible fluorescent marker is of great concern, since many published studies extending beyond 9 days have used this labeling method for evaluation of bacterial persistence [20],[21],[22]. In contrast, the comparable populations of enumerated rifampicin-resistant and rifampicin sensitive *E. coli* O157:H7 observed for the chromosomally marked strain indicate that this labeling method may better represent the actual cell population for the duration of the study.

Genetic differences can result in differences in plasmid segregation and differences in the rate of emergence of plasmid-free mutants [23]. Thus, rapid loss of the plasmid from tested *E. coli* O157:H7 may not be representative of all *E. coli*, but these experiments demonstrate that rapid loss occurs in some strains. If researchers choose to label *E. coli* O157:H7 for experimental purposes, it would be prudent to assess the stability of the specific plasmid in the unique bacterial host prior to launching long-term experiments. Likewise, because of possible differences in the genomic background of the strains used for the GFP survival and rifampicin labeling, we refrained from making direct comparisons between the utility of these markers.

In this study, the evaluation of the growth kinetics for the GFP-labeled *E. coli* O157:H7 and the chromosomally labeled *E. coli* O157:H7 indicate that neither labeling procedures altered the short-term growth characteristics of the organisms under the laboratory conditions studied. The presence of the plasmid did not place a significant metabolic burden on the cell. This finding is consistent with previously published data which also demonstrated the presence the GFP plasmid did not affect the growth kinetics of the bacteria under optimal growth conditions [5],[24],[25],[26].

Plasmid loss can be caused by either structural or segregational instability [23]. Structural instability occurs as a result of loss of the DNA sequence during replication or recombination. Segregational instability results in the failure of the plasmids to be distributed between the two daughter cells thus resulting in the evolution of cells that are plasmid free. Segregational instability can be mediated by nutrient availability, growth rate, host and cellular genotype [27],[28]. This underscores the need to understand the stability of the plasmid in the environment to be tested prior to conducting survival studies. Variations in survival times in different matrices measured using GFP-labeled cells may be artifacts of plasmid stability instead of bacterial survival in general.

Spontaneous rifampicin resistance is typically conferred by mutations in the RNA polymerase gene [29]. As such, it is possible that this mutation or compensatory mutations may affect bacterial growth and or survival. The results obtained for the rifampicin resistant *E. coli* O157:H7 in this study demonstrated that the chromosomal mutation did not inhibit the growth of the organism over the time period tested. The use of homologous recombination to insert antibiotic resistance genes into known locations in the genome may provide another alternative to confer a phenotypic marker to *E. coli* O157:H7.

While GFP labeling of bacterial cells has many advantages, one has to be conscious of the potential instability of the plasmid maintenance in long-term survival experiments. This experiment demonstrated that once the selective pressure had been removed, plasmid loss and subsequent loss of phenotypic labeling characteristics occur in nutrient-deficient matrices.
